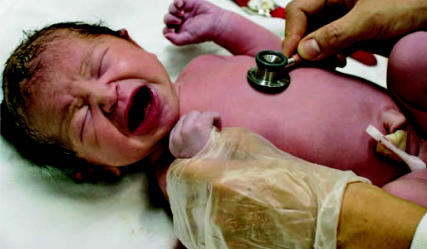# Shift in the Sexes: Are Endocrine Disruptors Changing Birth Ratios?

**Published:** 2007-06

**Authors:** Julia R. Barrett

According to demographic data compiled by the United Nations, an average of 105 boys are born for every 100 girls. The male proportion of births, equal to 0.515, varies slightly between years and populations, but these factors do not fully explain consistently shifting ratios in several industrialized countries over recent decades. A new study examines birth and fetal death sex ratios in Japan and the United States and reveals significant male-to-female shifts in both nations **[*EHP* 115:941–946; Davis et al.]**.

The research team calculated birth and fetal death sex ratios in Japan based on 1949–1999 data from the Japanese Vital Statistics Bureau. The proportion of male births varied yearly before 1970 but declined steadily since then, from 0.5172 to 0.5135. Between 1960 and 1999, the male proportion of fetal deaths increased from 56% to 67.7%. The male fetal death rate is approximately four times higher in Japan than in the United States.

For U.S. calculations, the researchers drew 1983–1995 fetal death data and 1970–2002 birth data from the National Center for Health Statistics. The proportion of male births dropped in the United States, from 0.5134 in 1970 to 0.5117 in 2002. There are significant racial differences, however: between 1970 and 2002 the proportion of non-Hispanic white male births fell from 0.5143 to 0.5122, whereas the proportion of black male births rose slightly from 0.5076 to 0.5079. The male proportion of black fetal deaths also increased, rising from 53.5% to 54.5%; among whites, the male proportion of fetal deaths rose by less than 0.5%.

Why birth sex ratios differ so much between white and black women is unknown, but hormonal differences due to race and to incidence of obesity may be involved. A possible explanation for the increased ratio among black births may stem from improved prenatal and obstetric care in general, reducing the overall number of fetal deaths.

The researchers speculate that parental exposures to endocrine-disrupting chemicals, including metalloestrogens such as methylmercury, might be factors undermining the conception and survival of male children. They suggest particular scrutiny of Japanese body burden of mercury and other metalloestrogens to understand this difference. Additionally, future investigations of declining sex ratios should consider the types and timing of prenatal and parental exposures to endocrine-disrupting chemicals. The researchers hypothesize that paternal exposures prior to conception might affect expression of the *SRY* gene on the Y chromosome.

## Figures and Tables

**Figure:**